# Developing an Active Biodegradable Bio-Based Equilibrium Modified Atmosphere Packaging Containing a Carvacrol-Emitting Sachet for Cherry Tomatoes

**DOI:** 10.3390/foods13213371

**Published:** 2024-10-23

**Authors:** Anastasia E. Kapetanakou, Antonis Mistriotis, Dimitra C. Bozinaki, Philippos Tserotas, Ioanna-Georgia Athanasoulia, Demetrios Briassoulis, Panagiotis N. Skandamis

**Affiliations:** 1 Laboratory of Food Quality Control and Hygiene, Department of Food Science and Human Nutrition, Agricultural University of Athens, Iera Odos 75, 11855 Votanikos, Greecepskan@aua.gr (P.N.S.); 2 Institute of Technology of Agricultural Products, Hellenic Agricultural Organization-DIMITRA, S. Venizelou 1, 14123 Lycovrissi, Greece; 3 Laboratory of Farm Structures, Department of Natural Resources Management and Agricultural Engineering, Agricultural University of Athens, Iera Odos 75, 11855 Votanikos, Greecebriassou@aua.gr (D.B.)

**Keywords:** active sachet, micro-perforation, bio-based biodegradable packaging, carvacrol, cherry tomatoes, shelf-life

## Abstract

This study aimed to develop an active biodegradable bio-based (polylactic acid/PLA) equilibrium modified atmosphere packaging (EMAP) containing a carvacrol-emitting sachet (created by poly-hydroxybutyrate) (PLA-PHB-CARV) to extend the shelf-life of cherry tomatoes at 15 °C and 25 °C. Cherry tomatoes in macro-perforated polypropylene (PP) films (mimicking the commercial packaging) or in PLA-based micro-perforated film without the carvacrol sachet (PLA) were also tested. Weight loss, decay, headspace gases, pH, titratable acidity (TA), total suspended solids (TSS), ripening index, color, texture, total viable counts (TVC), and sensory analysis were performed. Decay was 40% in PLA-PHB-CARV, and 97% in PP after 20 days at 25 °C. PLA-PHB-CARV showed lower weight loss (*p* < 0.05) and stable firmness compared to PP and PLA at both temperatures. TSS and TA were not affected by the packaging at 15 °C, while at 25 °C, the TSS accumulation was inhibited in PLA-PHB-CARV compared to in PLA and PP (*p* < 0.05), indicating a notable delay in the ripening process. PLA-PHB-CARV retained their red color during storage compared to PP and PLA. Carvacrol addition inhibited TVC compared to PP and PLA by ca. 2.0 log CFU/g during storage at 25 °C, while at 15 °C, the packaging did not reveal a significant effect. Overall, the results indicated that the developed active EMAP may be adequately used as an advanced and alternative packaging for tomatoes or potentially other fruits with a similar respiration rate versus their conventional packaging, showing several advantages, e.g., a reduction in petrochemical-based plastics use, shelf-life extension of the packaged food, and consequently, the perspective of limiting food waste during distribution and retail or domestic storage.

## 1. Introduction

Tomato (*Solanum lycopersicum* L.) is a climacteric berry fruit with an increased post-harvest respiration rate and ethylene bursts when non-properly controlled storage temperatures occur, or under high relative humidity (RH) storage conditions, thus leading to a high volume of post-harvest losses, which constitutes a remarkable economic burden for the fresh produce industry [1]. The global tomato market size was valued at USD 174.7 billion in 2023, while it is expected to reach USD 233.13 billion in 2028, at a compound annual growth rate (CAGR) of 5.7% during the forecast period [2]. Cherry tomatoes are among the most popular high-value crops due to their nutrient content and taste, while they constitute a great healthy and convenient snack when packaged accordingly. Considering the above, along with the increasing demand from consumers, a significant boost in cherry tomato cultivation is expected in the coming years.

Packaging may significantly contribute to cherry tomatoes’ production value chain in diverse ways, i.e., providing vital protection against physical, chemical, and/or biological contaminants, prolonging products’ shelf-life, serving as a powerful marketing tool, enhancing consumers’ convenience, conveying important information to consumers, and ensuring traceability for the food company. Nowadays, cherry tomatoes are commercially distributed in clamshells, in bags (placed in paperboard trays) made of macro-perforated petrochemical-based plastics like polypropylene (PP), in wooden crates/boxes, or in corrugated cardboard boxes [3]. Specifically, petrochemical-based plastics are widely used by the food industry due to their advanced functional characteristics, i.e., mechanical and barrier properties against O_2_ and aroma compounds, as well as their heat sealability, low cost, and high availability. However, their environmental persistence (due to their slow degradation rate) is one of their main disadvantages, which in recent years has raised great ecological and public health concerns, prompting the emergence of a trend for their replacement by novel bio-based materials that are biodegradable under composting conditions, i.e., polylactic acid (PLA) or poly-hydroxybutyrate (PHB). Researchers have previously focused on developing sustainable packaging for tomatoes or other fruits using PLA or PHB films or trays, with promising results in terms of shelf-life extension compared to the conventional packaging means which are available on the market [4,5,6,7]. Moreover, several studies have revealed promising results by combining the use of PLA or PHB with an alternative packaging technique known as equilibrium modified atmosphere packaging (EMAP), where a modification of the packaging film’s permeability takes place, usually through perforation, aiming to optimally regulate and dynamically modify the in-package atmosphere of tomatoes, considering their strong climacteric character [8,9,10].

Active antimicrobial packaging refers to the emerging technology in which the packaging material interacts with the packaged food and/or the package headspace to maintain or improve the quality, safety, and sensory aspects of the preserved food, thus extending its shelf-life [11,12]. Specifically, there are two main forms of active antimicrobial packaging: (i) the direct incorporation of the antimicrobial compound into the polymeric matrix by coating onto the packaging surface; and (ii) the immobilization of a volatile antimicrobial on emitting pads or in sachets and placing inside the packaging [11,13]. Although the use of immobilized antimicrobial agents inside a polymeric matrix is a popular research topic due to the dual mode of action via migration and evaporation, the contact between the antimicrobial and the food product is a main drawback, since this may potentially result in undesirable effects like intense flavor or color alterations [14]. Similar limitations exist when emitting pads with antimicrobial substances are applied in active packaging, especially when the pad is placed under the product [15,16]. Moreover, the active substance does not diffuse or evaporate in a controlled way. On the other hand, active sachets, placed inside the packaging on the side or on the top of the packaging film (facing the headspace of the packaging), have generated great interest among the scientific community, since not only do they allow for the gradual and controlled release of the vapor phase of the antimicrobial compound in the packaging headspace, but also avoid any contact between the food and the antimicrobial. Specifically, previous studies have focused on developing emitting sachets with volatile antimicrobial/antioxidant compounds, with a special focus on essential oils (EOs) and/or their main active components due to their multiple beneficial actions, to assess their potential for extending the shelf-life of tomatoes or other various plant products such as avocadoes, fresh case gooseberries, or mushrooms [16,17,18,19,20].

A new research area in food packaging is to assess the perspective of combining the above alternative packaging techniques (EMAP or active packaging) along with the use of biodegradable bio-based materials, thus contributing to the development and use of sustainable packaging solutions, as well as the minimization of food waste via the shelf-life extension of the food product. Specifically, Sun et al. (2024) [7] developed an EMAP and PLA-based packaging for “Kyoho” grapes, showing increased quality maintenance in several parameters like weight loss, decay, vitamin C, total phenolics, and total anthocyanins compared to controls during storage at 5 °C for 35 days. Similarly, Garavito et al. (2022) [19] configurated EMAP-PLA trays for fresh case gooseberries, which revealed an extended shelf-life (42 days) at 6 °C compared to the unpackaged product (21 days). Moreover, studies have successfully shown the antimicrobial/antioxidant effect of bio-based active packaging on cherry tomatoes by applying, i.e., PHB film with grapeseed oil and MgO nanoparticles or PLA films incorporating orange peel extract [6,21]. Considering the above, the present study aimed to combine both techniques, EMAP and active packaging, along with the use of bio-based materials in order to develop active and fully biodegradable packaging for climacteric fruit like cherry tomatoes as an alternative to the existing conventional packaging. Specifically, throughout the current study, an active PLA-based EMAP containing a carvacrol-emitting sachet (created by PHB) was assessed for its potential to extend the shelf-life of cherry tomatoes (var. Acorn) by monitoring a series of quality parameters as well as the growth of indigenous microbiota during storage at 15 °C and 25 °C for up to 40 days.

## 2. Materials and Methods

### 2.1. Preparation of Cherry Tomatoes

The commercial packages (250 g) of cherry tomatoes (*Solanum lycopersicum*; var. Acorn) in macro-perforated plastic containers were purchased from a local supermarket (Athens, Greece) in September 2021 and transported to the laboratory. To minimize sample variation, fruits from the same lot were used. The packages were opened to remove fruits with bruises, wounds, or other disorders and only healthy cherry tomatoes were used. The handling and preparation of cherry tomatoes took place under aseptic conditions. Cherry tomatoes of ca. 300 ± 2 g (35–40 pieces per package) were placed in series inside a food contact paper kraft tray (22 × 16 × 6 cm), covering ca. 2/3 of the tray’s bottom surface and stored at 2–4 °C for max. 1 h. Paper kraft trays were sterilized by ultraviolet overnight before use.

### 2.2. Preparation of Carvacrol-Emitting Sachets

PHB was selected as the suitable bio-based, biodegradable material to create the carvacrol-emitting sachets for a series of specific reasons: (i) PHB is a bio-based polymer that can directly be produced by microorganisms through the bacterial fermentation of food industry waste and stored in their cells as a form of energy storage molecule [22]. (ii) The polymer easily biodegrades in many environments, such as soil, compost, freshwater, and seawater in contrast to other bio-based polymers, such as PLA, which only biodegrades in industrial compost environment [23]. Specifically, the PHB sachets prepared and used in the current study were tested for their biodegradation in soil, and according to the results, they biodegraded in less than 1 year, reaching an average biodegradation degree of 96%. In fact, PHB remained biodegradable in the soil, even being in contact with the studied bioactive compound (carvacrol). (iii) PHB, as the covering material of the sachets, offered satisfactory barrier properties for carvacrol vapors, allowing for its controlled release only through appropriately designed perforation. (iv) PHB produced by the fermentation of food waste contributes to the integration of the food industry into the circular economy.

To create carvacrol-emitting sachets, PHB films produced in the laboratory were cut into dimensions of 2 × 4 cm and half of them were micro-perforated with a homemade tool capable of simultaneously creating 14 holes of 500 μm diameter each, while the other half remained intact. Each PHB sachet was created by heat-sealing 3 out of 4 sides of one micro-perforated PHB film and one intact PHB film using a packaging machine LERICA C312 (Musile di Piave, Italy). Aliquot of 5 μL of carvacrol was added in a cellulose-absorbent pad, and immediately after, the pad was placed inside the sachet and heat-sealed. Cellulose absorbent pads were selected, since cellulose as a material has a series of fit-for-purpose advantages, namely being bio-based, biodegradable, having high porosity—and thus a high absorption capacity—as well as being widely available [24]. The carvacrol-carrying pads were inserted into PHB sachets to avoid any direct contact between the antimicrobial agent with the produce, which could affect the taste. In this way, the packaged cherry tomatoes were only exposed to the controlled release of carvacrol vapors throughout storage. The quantity of 5 μL carvacrol per sachet was calculated whilst taking into account that diffusion only takes place through the perforations of the sachet, since the permeability of PHB to carvacrol is low. The permeability of the perforations was calculated following the modified Fick’s law [25]. The quantity of 5 μL carvacrol was found to be sufficient in order for the headspace of the package to be saturated with carvacrol vapors for the full duration of the experiment, despite the gradual loss of part of the carvacrol vapors through the perforations of the packaging film. Carvacrol is approved as an active substance by the European Commission [26].

### 2.3. Design of Active PLA-Based EMAP for Cherry Tomatoes

PLA is a bio-based compostable polymer produced through the polymerization of lactic acid. Lactic acid is industrially synthesized by the bacterial fermentation of carbohydrates of processed corn or other naturally produced carbohydrates [21]. PLA is the first polymer based on renewable raw materials which has been commercialized at the industrial scale and is currently used for short shelf-life products, while at the same time meeting the specifications of international standards [27]. It has been proven to be particularly efficient as a packaging material for EMAP systems for fresh vegetables and fruits [5,7,8], since it exhibits high water vapor permeability. In contrast, its permeability to other gases relevant to EMAP design, such as CO_2_ and O_2_, is comparable to conventional films such as PP. Exploiting this selective gas permeability of PLA, perforation patterns for EMAP based on PLA films can be designed to only regulate CO_2_ and O_2_, while water vapor transmission is controlled by the surface area of the packaging film. Therefore, the use of PLA films allows for an optimal design of EMAP for many fresh products. The number and diameter of perforations are designed to match the respiration behavior of tomatoes, and the area of the packaging film is selected to regulate the RH in the headspace considering the transpiration activity of the packaged product.

**Figure 1:**
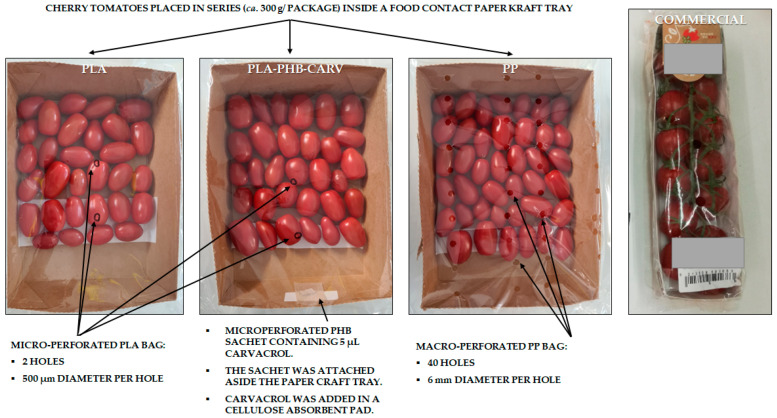
Food contact paper kraft tray with ca. 300 ± 2 g cherry tomatoes packaged in micro-perforated polylactic acid film (2 holes; 500 μm diameter each; indicated by the black circles) without (PLA) or with the addition of a micro-perforated PHB-sachet (14 holes; 500 μm diameter each) containing 5 μL carvacrol in a cellulose-absorbent pad (PLA-PHB-CARV) as well as placed in macro-perforated polypropylene bags (PP) (40 holes; 6 mm diameter each), mimicking the existing commercial packaging.

In the present study, two PLA-based EMAP configurations were designed, one with and one without the carvacrol-emitting sachet coded as PLA-PHB-CARV and PLA, respectively, as well as cherry tomatoes packaged in macro-perforated PP bags (40 holes of 6 mm diameter each) mimicking the commercial packaging (coded as PP) (Figure 1; Table 1). EMAP configurations were designed based on the methodology followed by Mistriotis et al. (2016) [8], who considered parameters like the respiration (5–8 mg kg^−1^ s^−1^) and transpiration flow rates (35–70 mg kg^−1^ s^−1^) of cherry tomatoes, while the targeted in-package RH and CO_2_ molar fraction were 80–90% and 3–4%, respectively. Previous researchers have highlighted that these ranges of RH and CO_2_ are close to the limits that ensure the lack of tissue injuries in most tomato cultivars [28]. Based on the aforementioned values, the authors of the present study calculated that 2 holes of 500 μm in diameter are sufficient to achieve the desired level of CO_2_ (3–4%) in an EMAP package for ca. 300 g of cherry tomatoes. Considering that the size of cherry tomatoes varies, differences in the number of fruits per package were expected. However, such variations are too small to have a significant effect on EMAP design. The size of the PLA bag used for the EMAP packaging was 30 × 24 × 6 cm (Table 1). Based on the existing data regarding the transpiration activity of cherry tomatoes and the water vapor transmission rate of PLA films, the size of the PLA bag, which does not differ much from the commercial PP packaging bag for the same quantity of cherry tomatoes, was calculated to increase the in-package RH to about 90%, thus reducing weight loss in comparison to unpackaged produce but also avoiding condensation. In the case of PLA-PHB-CARV samples, the micro-perforated side of the carvacrol-emitting PHB sachet (§ 2.2) was placed on the small side of the tray using a double-side adhesive tape (the sachet was added immediately after its preparation according to § 2.2 to avoid any carvacrol vapor losses), facing the headspace of the packaging without coming into contact with the cherry tomatoes. Immediately after placing the active sachet, each tray was packaged in a micro-perforated PLA bag (Figure 1). All PLA bags were sealed using a LERICA C312 (Musile di Piave, Italy) packaging machine and samples were stored in high-precision incubation chambers (±0.5 °C) (MIR-153, Sanyo Electric Co., Osaka, Japan) at 15 °C, mimicking the average storage temperature grocery corners placed within supermarkets (which is lower than room temperature, but still significantly above the refrigerated temperature range) and at 25 °C, mimicking the average storage temperature in open space grocery shops for up to 40 days. Although both studied temperatures are significantly higher compared to those in the refrigerated temperature range (ca. 4 °C), the authors hypothesized that differences would also occur in the results (per studied quality parameter and microbial growth), since the carvacrol release rate is highly dependent on the temperature. Specifically, previous studies have shown that the accumulated carvacrol release rate varied significantly between the temperatures of 4 °C, 25 °C, and 40 °C and, in fact, the release rate was significantly lower at 4 °C and 25 °C compared to that at 40 °C [29]. Two trays per packaging assay were prepared for each sampling day, while two independent storage experiments were performed (n = 4).

### 2.4. Headspace Gas Analysis

The headspace atmosphere composition in each package of cherry tomatoes was monitored using a portable PBI Dansensor A/S (Check Mate 9900 O_2_/CO_2_; Ringsted, Denmark) analyzer (accuracy ± 0.1%) with O_2_ and CO_2_ sensors [10]. Three (3) mL of gases from the package headspace was automatically sampled by the gas analyzer with a medical type of needle. The needle was inserted into the headspace via the existing holes of the films. Results were expressed as % O_2_ and % CO_2_.

### 2.5. Weight Loss

The initial and the final weight of each package was measured using an electronic weighing balance (Kern & Sohn GmbH; Balingen, Germany). Weight loss was calculated according to the following equation [6,17]:

(1) $$\%WL=\frac{W_{0}-W_{s}}{W_{0}}\times 100$$

where WL is the weight loss (%), W_0_ is the initial weight (g) on day 0, and W_s_ is the measured weight (g) of each sample at each sampling point.

### 2.6. Decay

Each packaging was carefully opened and macroscopically examined at regular time points to detect cherry tomatoes with visible decay, i.e., discoloration, shrinkage, tissue softening, or the presence of fungi. Decay was calculated by the following equation [1]:

(2) $$\%\;\mathrm{Decay}=\frac{Infected\;tomatoes}{Total\;number\;of\;tomatoes}\times 100$$

### 2.7. PH, Total Soluble Solids, Titratable Acidity, and Ripening Index

At each sampling point, 7–8 cherry tomatoes were randomly selected per package and their juice was extracted using a kitchen blender. Aliquots of 40 ± 10 mL of the tomato juice was used for the estimation of pH, total soluble solids (TSS), and titratable acidity (TA) [6,14,30].

Specifically, the pH of the tomato juice was measured using a digital pH meter (pH 526, Metrohm Ltd.; Herisau, Switzerland), while TSS were determined via a portable refractometer (Atago Co., Ltd.; Tokyo, Japan) at room temperature and the results were expressed in °Brix. TA was expressed as % citric acid (g of citric acid in 100 mL of tomato juice) after titration with NaOH 0.1 N. Finally, the ripening index (RI) was calculated by the equation:

(3) $$\%\;\mathrm{RI}=\frac{TSS}{TA}\times 100$$

### 2.8. Texture

The maximum force (F_max_) required to penetrate a cherry tomato was measured with a Texture Analyzer (Instron Series 5900; Norwood, MA, USA) with a 3.2 mm diameter cylindrical probe. A test speed of 20 mm min^−1^ and a distance of 8 mm were used. Firmness was expressed as the maximum penetration force (N), while the extension at maximum penetration force was also recorded. An average of five randomly selected cherry tomatoes of similar size was measured per sampling point and per package.

### 2.9. Color

Skin color of cherry tomatoes was evaluated by measuring the L* (lightness), a* (redness), and b* (yellowness) parameters (CIE L*a*b* color scale) using a portable spectrocolorimeter (Lovibond LC 100, Tintometer Inc.; Dortmund, Germany). Before the measurements, the colorimeter was calibrated with a white reference plate (L = 100). The color difference with true red was used for the color assessment of cherry tomatoes, which, according to previous studies [31], could be used as an objective ripening index, which may provide a realistic estimation of consumers’ perception:

(4) $$Color\;difference\;with\;true\;red=\sqrt{{\left(L*-50\right)}^{2}+{\left(a*-60\right)}^{2}+{\left(b*\right)}^{2}}$$

where L* is the total light reflected ranging from 0 (black) to 100 (white); a* is red (positive values) and green (negative values) colors; b* are yellow (positive values) and blue (negative values) colors. An average of five randomly selected cherry tomatoes per package was measured per sampling point, taking 10 readings per sample.

### 2.10. Sensory Evaluation

The sensory evaluation of cherry tomatoes was carried out on days 7 and 20 at 15 °C and on days 20 and 40 at 25 °C, in artificial light and at ambient temperature. Ten panelists belonging to the laboratory staff of Laboratory of Food Quality Control and Hygiene participated and blindly scored cherry tomatoes per packaging for color, odor, texture, and freshness. Each attribute was assessed on a hedonic scale from 1.0 (unacceptable–spoiled) to 3.0 (acceptable–fresh) with 0.5 intervals. A score of 2.0 indicated that cherry tomatoes change from typical fresh to not fresh; however, they were considered still acceptable.

### 2.11. Growth of Indigenous Microbiota

At each sampling point, the appropriate number of cherry tomatoes was randomly and aseptically removed from the packages to weight ca. 10 g mixed with 90 mL of sterile Ringer’s solution (Lab M Ltd.; Manchester, UK), and homogenized in a stomacher (Interscience; Paris, France) for 60 s. Following homogenization, decimal dilutions in Ringers’ solution were prepared and 0.1 mL of the appropriate dilutions were spread on Plate Count Agar (PCA) (Lab M Ltd.; Manchester, UK) and incubated at 30 °C for 48 h to enumerate the total viable counts (TVC). Results were expressed as log CFU/g of cherry tomatoes.

### 2.12. Statistical Analysis

Statistical analysis was performed with an SPSS computer package Version 16.0 (SPSS Inc., Chicago, IL, USA). The analysis of variance (ANOVA) was performed to evaluate the effect of packaging type, temperature, storage time, and their interactions on the quality parameters and microbial levels. Tukey’s b multiple range tests and *t*-tests were used for a mean comparison at the 95% significance level.

## 3. Results and Discussion

### 3.1. Headspace Gas Analysis

**Figure 2:**
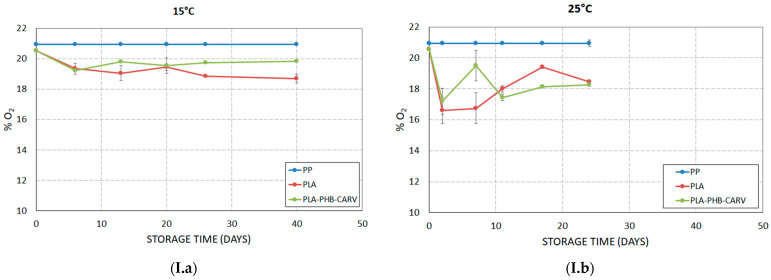

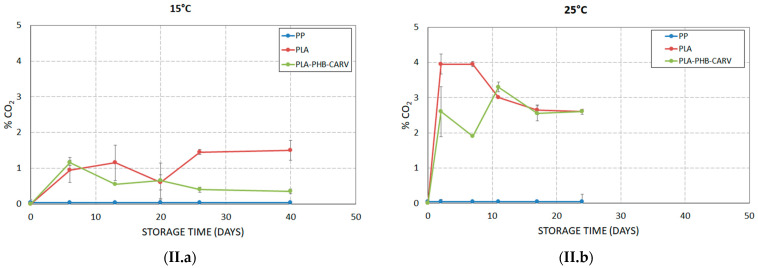
Evolution of (**I**) % O_2_ and (**II**) % CO_2_ on cherry tomatoes placed in paper kraft trays and packaged in PLA-based EMAP with (PLA-PHB-CARV) or without (PLA) a carvacrol-emitting sachet as well as in macro-perforated polypropylene (PP) bags during storage at (**a**) 15 °C and (**b**) 25 °C.

The composition of headspace gases inside the package depends on a series of factors, i.e., the permeability of packaging materials, the respiration rate, the ethylene production, and storage temperature [32]. Figure 2 shows the variation of headspace gas composition in % O_2_ and % CO_2_ during storage at 15 °C and 25 °C. For PP samples at both temperatures, the % O_2_ and % CO_2_ in the packaging was close to those of atmospheric air (O_2_ ≈ 20.95% and CO_2_ ≈ 0.038%) due to the macro-perforation of the packaging (40 holes of 6 mm diameter) (Figure 1 and Figure 2; Table 1). Gas measurements in tomatoes packaged in PLA with or without the carvacrol-emitting sachet at 25 °C revealed an increase in CO_2_ concentration from 0% to ca. 3–4% during the first 2 days of storage, while thereafter, % CO_2_ was maintained to an average value of ca. 3% (Figure 2(II.b)), which was expected according to the authors’ calculations when the PLA-based EMAP was originally designed (CO_2_ target values: 3–4%) (§ 2.3). A similar trend of % CO_2_ accumulation was recorded during the 40 days of storage at 15 °C; however, the maximum recorded values in PLA and PLA-PHB-CARV were lower, namely 0.5–1.5% (reached on day 6 and remained until the end of the storage) (Figure 2(II.a)). Moreover, a slight but significant (*p* < 0.05) inhibition of % CO_2_ evolution was recorded in PLA-PHB-CARV compared to PLA samples, at both storage temperatures (Figure 2(II.a),(II.b)), indicating that the presence of carvacrol may delay the respiration rate of fruits. Previous researchers have reported contradictory results, showing that EO vapors may either delay or stimulate respiration in tomatoes depending on factors like the applied EO’s concentration and fruit’s maturation stage [33,34]. Regarding % O_2_, a restricted but significant decrease (*p* < 0.05) was recorded in PLA and PLA-PHB-CARV samples from 21% to ≈ 18% at 15 °C (on day 40) and from 21% to 16–18 % at 25 °C (on day 24) (Figure 2(I.a),(I.b)). Comparing the two storage temperatures, it is obvious that the respiration was more intense at 25 °C compared to 15 °C, an a priori expected and well-reported result. In fact, previous researchers have highlighted that, when tomatoes are stored at temperatures < 20 °C, many metabolic activities, transpiration process, and water loss are retarded, thus extending their shelf-life [35].

### 3.2. Decay

**Figure 3:**
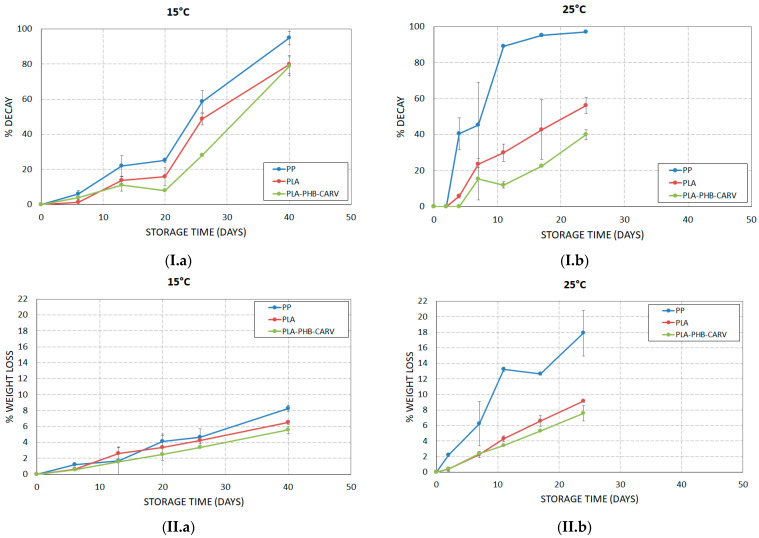
Variation of (**I**) % decay and (**II**) % weight loss on cherry tomatoes placed in paper kraft trays and packaged in PLA-based EMAP with (PLA-PHB-CARV) or without (PLA) carvacrol-emitting sachet as well as in macro-perforated polypropylene (PP) bags during storage at (**a**) 15 °C and (**b**) 25 °C.

The results showed that the % decay in cherry tomatoes followed the order of PP > PLA > PLA-PHB-CARV, regardless of the storage temperature (*p* < 0.05) (Figure 3(I.a),(I.b)). Specifically, at 25 °C, PP showed the highest decay rate, reaching 97% on day 24 (over 85% of tomatoes were spoiled even from day 11), while, at the same time, tomatoes packaged in PLA without and with the active emitting sachet showed a corresponding rate of 56% (PLA) and ≈ 40% (PLA-PHB-CARV), respectively (Figure 3(I.b)). A similar inhibitory trend (*p* < 0.05) was also observed in PLA and PLA-PHB-CARV packaged samples compared to PP during storage at 15 °C, yet at a lower rate and extent than 25 °C (Figure 3(I.a)). In fact, although the percentage of spoiled tomatoes on day 40 was similar (≈ 80%) in PLA and PLA-PHB-CARV, it is obvious that the application of a carvacrol-emitting sachet caused an additional significant inhibition on the decay rate (*p* < 0.05) (Figure 3(I.a)). The inhibitory effect of the active PLA-based EMAP on the decay of cherry tomatoes is directly related to the in-package atmosphere. For example, the notably lower in-package % O_2_ (ca. 16–20%) in PLA samples compared to the almost aerobic conditions existing in PP samples during storage at 25 °C (Figure 2(I.b)) caused the suppression of several metabolic activities. The latter result is in agreement with the findings of previous researchers [8], who reported a decay of ca. 5% for cherry tomatoes packaged in PLA compared to the recorded ca. 11% in PP during storage at 20 °C. The inhibitory effect of carvacrol on the currently observed % decay of carvacrol-packaged tomatoes has previously been reported by several researchers. Buendia-Moreno et al. (2019) [36] recorded that the percentage of spoiled tomatoes in an active packaging having an inner coating of nano-encapsulated carvacrol in β-cyclodextrin was lower (ca. 1%) compared to controls (ca. 7%) after 24 days at 8 °C. Moreover, Shemesh et al. (2016) [37] showed that tomatoes packaged in macro-perforated polyamide-carvacrol bags revealed reduced decay development compared to the controls during storage at 10 °C for 51 days. Overall, the results of the present study suggested that the addition of the carvacrol-emitting sachet in the EMAP increased the shelf-life of cherry tomatoes, in terms of % decay, not only at 15 °C but also at the temperature of 25 °C compared to PP and PLA.

### 3.3. Weight Loss

**Figure 4:**
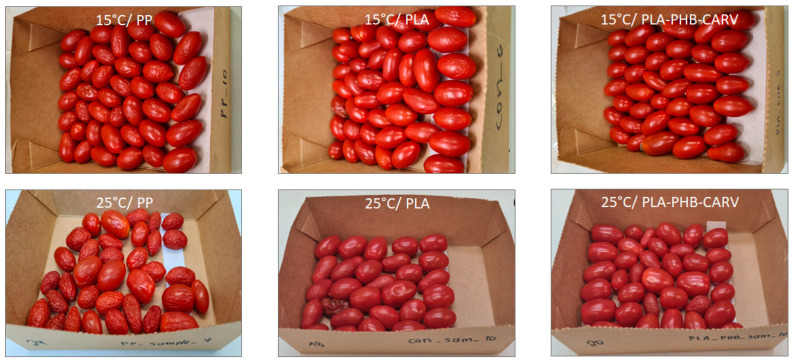
Comparative images of cherry tomatoes placed in paper kraft trays and packaged in PLA-based EMAP with (PLA-PHB-CARV) or without (PLA) a carvacrol-emitting sachet as well as in macro-perforated polypropylene (PP) bags stored at 15 °C (on day 40) and 25 °C (on day 24).

The results showed that % weight loss in cherry tomatoes followed the order: PP > PLA > PLA-PHB-CARV (*p* < 0.05) during storage at 25 °C (Figure 3(II.b)). Specifically, tomatoes packaged in PP recorded significant and rapid weight loss (*p* < 0.05) reaching 18.0 ± 3.0% on day 24, while the PLA and PLA-PHB-CARV samples revealed a weight loss of 9.2 ± 0.2% and 7.6 ± 1.0%, respectively (Figure 3(II.b)). This intense weight loss in PP compared to PLA and PLA-PHB-CARV samples (*p* < 0.05) was also macroscopically observed (Figure 4). All the above verify that the active PLA-based EMAP was efficiently designed to regulate the water exchange between the headspace and the external environment of the package. At 15 °C, the recorded % weight loss was at similar levels among the different packaging assays throughout storage (*p* ≥ 0.05) (Figure 3(II.a)). In fact, the weight loss remained ≤ 5% until day 26, which is the critical limit for the reduction in the market value of fruits and vegetables according to relevant reports [38]. On the contrary, after 40 days of storage, the recorded % weight loss reached a max. range of 5.6–8.3% regardless of the packaging type. Moreover, it is obvious that weight loss was significantly delayed by the reduction in temperature (*p* < 0.05) for all studied assays (especially in PP stored at 15 °C) (Figure 3(II.a),(II.b)). It is well known that weight loss in fresh produce during storage occurs due to the oxidation process, a phenomenon that becomes more evident in climacteric products (high respiration rate) such as tomatoes. Previous researchers have reported similar results when EOs were applied (encapsulated in a sachet or applied as a coating direct on the packaging) during the storage of tomatoes in a wide temperature range (8–25 °C) [17,36,39], which highlighted that EOs may act as a barrier to moisture loss due to their hydrophobic nature, thus allowing a good lipid partitioning of the cell membranes of the fruit peels. In summary, the result that PLA-PHB-CARV samples revealed the lowest % weight loss among the studied packaging shows that it was a component of the efficient regulation of water exchange between the in- and outside-package environment by the PLA-based EMAP and the barrier properties of carvacrol against moisture loss.

### 3.4. PH, Titratable Acidity, Total Soluble Solids, and Ripening Index

**Figure 5:**
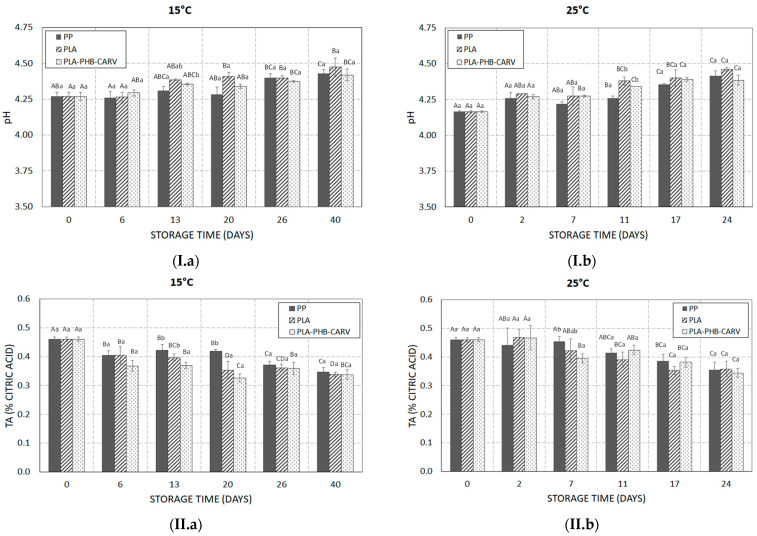

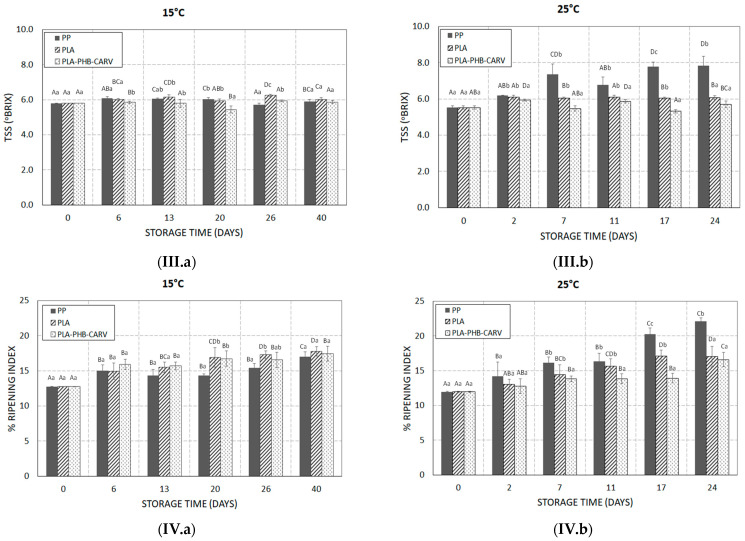
Variation in (**I**) pH, (**II**) titratable acidity (TA), (**III**) total soluble solids (TSS), and (**IV**) ripening index (%) for cherry tomatoes placed in paper kraft trays and packaged in PLA-based EMAP with (PLA-PHB-CARV) or without (PLA) a carvacrol-emitting sachet as well as in macro-perforated polypropylene (PP) bags during storage at (**a**) 15 °C and (**b**) 25 °C. Values in bars within the same storage day with different lowercase letters are significantly different (*p* < 0.05). Values in bars within the same packaging type with different uppercase letters are significantly different (*p* < 0.05).

Figure 5 shows the variation in (i) pH, (ii) TA (% citric acid), (iii) TSS (°Brix), and (iv) % RI of cherry tomatoes packaged with PP film vs. PLA film with or without the carvacrol-emitting sachet during storage at 15 °C and 25 °C. Specifically, at 25 °C, pH significantly increased (*p* < 0.05) from 4.17 ± 0.01 (on day 0) to 4.39–4.46 (on day 24) (Figure 5(I.b)), while TA decreased (*p* < 0.05) from 0.46 ± 0.01 (on day 0) to 0.34–0.36 (on day 24) (*p* < 0.05), regardless of the packaging type (Figure 5(II.b)). Similar significant increasing and decreasing trends (*p* < 0.05) were also recorded in pH and TA, respectively, during storage at 15 °C (Figure 5(I.a),(II.a)). The observed pH increase/TA decrease throughout storage is related to the ripening process, during which organic acids are used as an energy source for the respiration process [40]. According to Cantwell (2006) [41], in terms of tomato quality and freshness, the value of pH is expected to range from 4.17 to 4.59, something that was observed for all samples in the present study, regardless of the storage temperature and packaging type. The type of packaging seems not to play a crucial role (*p* ≥ 0.05), on the variation of pH or TA in most of the studied assays. The latter result differs from previous researchers, who reported a significant inhibitory effect of Eos’ vapors in the TA of tomatoes applied inside the packaging, either via a sachet containing the EO in a form of spray-dried emulsion powder or by placing the liquid EO in tubes compared to controls [34,39]. Potential reasons for such differences may be associated with the EOs’ type and concentration, or the type of encapsulation as well as the storage temperature, which may significantly affect the release rate of EO. Contrary to pH and TA, the packaging type played a significant role in TSS (*p* < 0.05), especially at 25 °C. Specifically, our results showed a significant increase in PP samples (from 5.5 ± 0.1°Brix to 7.9 ± 0.5°Brix) (*p* < 0.05) throughout storage at 25 °C (Figure 5(III.b)) compared to the limited variation observed in PLA samples (from 5.5 ± 0.1°Brix to 6.1 ± 0.1°Brix) and the recorded stability observed in PLA-PHB-CARV samples (from 5.5 ± 0.1°Brix to 5.8 ± 0.2°Brix) (Figure 5(III.b)). At 15 °C, TSS remained stable and close to the initial values (from 5.5 ± 0.1°Brix to 5.7 ± 0.2°Brix) during storage, notably suppressing the ripening process of cherry tomatoes, regardless of packaging type (Figure 5(III.a)). It is well known that TSS accumulation is directly related to the increase in sugar concentration due to the hydrolyzation of soluble polysaccharides into simple sugars during ripening [40]. The significant inhibition of TSS accumulation in the PLA and PLA-PHB-CARV samples may be related to the decrease in fruits’ respiration rate caused by the application of PLA-based EMAP compared to the almost existing aerobic conditions in PP samples (Figure 2). Many previous reports, who developed active packaging using the bioactive compounds of oregano EO (either carvacrol or thymol) for tomatoes, showed that the addition of EO, either via nano-encapsulation in β-cyclodextrin and coating on the cardboard trays, or through incorporation in a sachet, did not significantly affect the TSS accumulation [17,36,42]. Such differences could be related to the release rate of carvacrol among the different carriers of encapsulation, and the permeability of the packaging film. Regarding the % RI at 25 °C, PP samples showed a significant increase (*p* < 0.05) from 12% to 22%, while PLA and PLA-PHB-CARV samples revealed a restricted increase reaching after 24 days ≈ 17% (Figure 5(IV.b)). At 15 °C, although an increase in % RI was also recorded during storage, the differences between the packaging assays were less evident compared to 25 °C, however significant (*p* < 0.05) (Figure 5(IV.a),(IV.b)). Assessing the overall impact of the packaging type in all the above parameters, it seems that PLA-based EMAP with a carvacrol-emitting sachet significantly (*p* < 0.05) delayed the cherry tomatoes ripening, a result that was also macroscopically observed (Figure 4).

### 3.5. Texture

**Figure 6:**
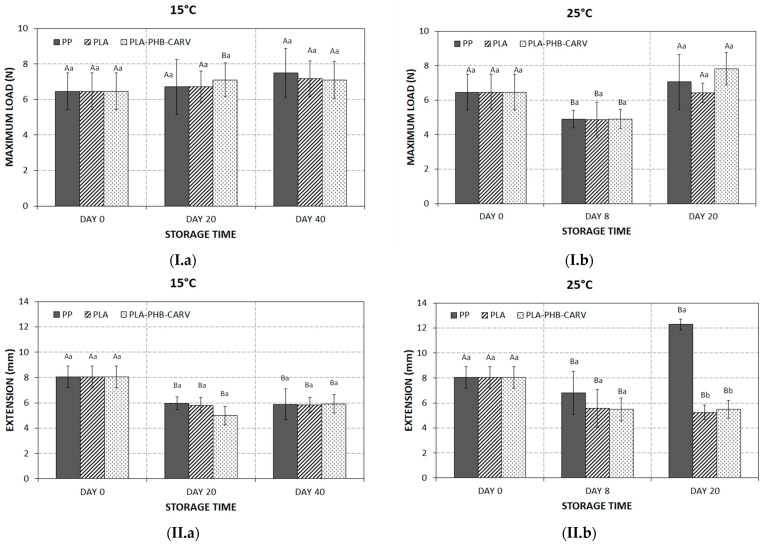
Variation of (**I**) penetration force (F_max_; N) and (**II**) extension (mm) at penetration point on cherry tomatoes placed in paper kraft trays and packaged in PLA-based EMAP with (PLA-PHB-CARV) or without (PLA) a carvacrol-emitting sachet as well as in macro-perforated polypropylene (PP) bags during storage at (**a**) 15 °C and (**b**) 25 °C. Values in bars within the same storage day with different lowercase letters are significantly different (*p* < 0.05). Values in bars within the same packaging type with different uppercase letters are significantly different (*p* < 0.05).

It is well known that fruit texture is subject to post-harvest changes, namely during ripening and storage, due to molecular and biochemical processes that cause the breakdown and deterioration of cell membrane, cell wall composition, intracellular materials, and overall cell structure [38]. In the present study, the maximum penetration force and the extension at penetration point was monitored to assess how texture alters among the different packaging types of cherry tomatoes. Specifically, the F_max_ of all samples remained close to the initial values (6.46 ± 1.04 N) during storage at 15 °C (*p* ≥ 0.05) (Figure 6(I.a)). The extension at the penetration point showed a significant decreasing trend (*p* < 0.05) regardless of packaging type (Figure 6(II.a)), indicating that the tomatoes’ surfaces became crispier during storage. Moreover, assessing the results between day 0 and day 20 at 25 °C, no significant differences were recorded on F_max_ for any of the packaging types (Figure 6(I.b)). With regard to the extension recorded at the penetration point, cherry tomatoes packaged in PP revealed a significant increase (from 8.05 ± 0.86 mm on day 0 to 12.28 ± 0.46 mm on day 20) (*p* < 0.05) (Figure 6(II.b)), indicating that the tomatoes became softer and acquired more elasticity due to the recorded high % weight loss and visual shriveling (Figure 3(II.b); Figure 4), caused by the temperature of 25 °C and macro-perforation of PP (Table 1; Figure 1). Previous researchers have highlighted that the surfaces of tomatoes soften due to the production of ethylene, a volatile metabolite which gradually accumulates in the packaging’s headspace during ripening, turgor loss, and/or the enzymatic breakdown of the pectin moiety within the product tissue by pectolytic enzymes, which is especially enforced at abuse temperatures [40,43,44]. On the other hand, the PLA and PLA-PHB-CARV samples showed significantly (*p* < 0.05) lower values of extension at the penetration point and stable F_max_, indicating that their texture remained close to the initial values, even during storage at 25 °C (Figure 6(I.b),(II.b)). Compared with previous researchers, who developed active packaging using EOs or their bioactive compounds, Buendia-Moreno et al. (2019) [42] reported that tomatoes packaged in an active tray with an inner coating of nano-encapsulated mix of oregano EO–cinnamon EO–carvacrol in β-cyclodextrin retained their unchanged firmness (*p* > 0.05) compared to the controls, where firmness was reduced by 2.5 N during storage at 8 °C for 24 days. On the other hand, researchers have also indicated that EOs may damage the plant cells of horticultural products, reducing the product quality and consequently affecting consumer acceptance, a result that is, however, dependent on the EOs’ concentration [45]. Overall, the results showed that PLA and PLA-PHB-CARV samples retained a stable texture for a longer storage time compared to the PP samples, even at 25 °C; however, the application of carvacrol (PLA-PHB-CARV) did not have an additional positive effect compared to PLA, highlighting the crucial role of PLA-based EMAP in texture. Specifically, previous studies has shown that the modification of an in-package atmosphere may slow down the process of ripening, which is strongly linked to the evolution of firmness. In fact, it has been demonstrated that a modified atmosphere decreases the activity of pectolytic enzymes, such as pectinesterase and poligaracturonase enzymes involved in the cell wall degradation [46,47].

### 3.6. Color

Color in tomatoes is the most important visible characteristic used to assess ripening and post-harvest life, and it is a major criterion for consumers during purchase. As reported in §2.9, the color difference with true red was selected as the objective ripening parameter providing a realistic estimation of consumers’ perception (Table 2). At 25 °C, the PP samples had the greatest and fastest decrease (*p* < 0.05) (from 46.46 ± 2.28 to 43.39 ± 2.44) in color difference with true red during the 24-day storage compared to the stability observed in PLA (from 46.46 ± 2.28 to 46.15 ± 3.75) and PLA-PHB-CARV samples (from 46.46 ± 2.28 to 46.62 ± 2.78). At 15 °C, the color difference with true red on cherry tomatoes showed a gradual significant decrease (*p* < 0.05) throughout the storage period of 40 days, not only in PP but also in PLA-based EMAP with or without carvacrol sachet; however, these differences were not macroscopically visible (Figure 4). Moreover, according to the statistical analysis, the monitored index showed that the packaging type did not play significant role (*p* ≥ 0.05) on the color of cherry tomatoes during storage at 15 °C (Table 2). Regarding PP, the higher color changes recorded at 25 °C were expected compared to 15 °C due to the faster temperature-related ripening process (Figure 5(IV.a),(IV.b)). As is well known, lycopene is responsible for the red color of tomatoes, which is formed from the colorless precursor phytoene during chlorophyll degradation [48]. It seems that the controlled atmosphere that PLA-based EMAP offered to the packaged cherry tomatoes along with the antioxidant properties of carvacrol may delay the degradation of lycopene, thus preserving the red color of tomatoes. In fact, several researchers have showcased the adequate preservation of tomatoes’ red color via spectro-colorimeter or by quantifying the lycopene concentration, when vapors of various EOs (i.e., oregano, pink pepper, cinnamon) or their bioactive compounds (i.e., carvacrol, thymol) were directly applied in the packaging either via sachet or coating on the tray or the packaging film [17,36,39].

### 3.7. Sensory Evaluation

**Figure 7:**
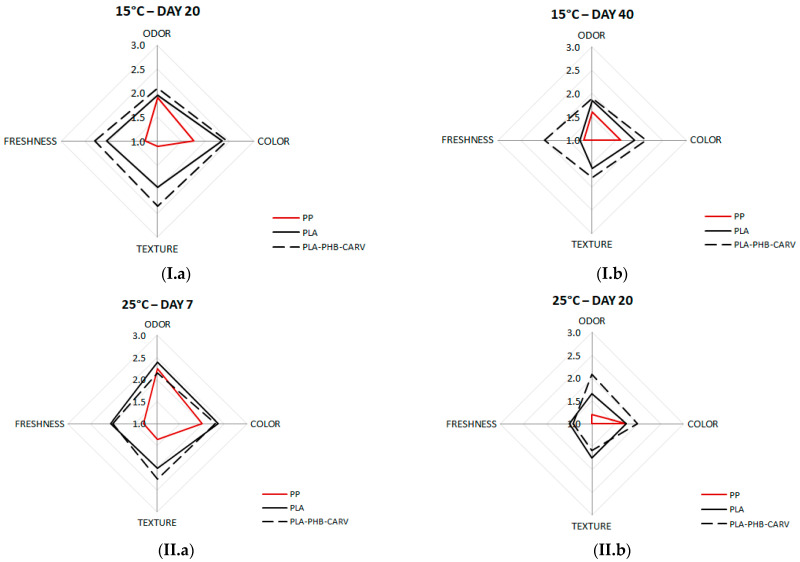
Sensory evaluation of odor, color, texture, and freshness of cherry tomatoes placed in paper kraft trays and packaged in PLA-based EMAP with (PLA-PHB-CARV) or without (PLA) a carvacrol-emitting sachet as well as in macro-perforated polypropylene (PP) bags during storage (**I**) at 15 °C (**a**) on day 20 and (**b**) on day 40 and (**II**) at 25 °C (**a**) on day 7 and (**b**) on day 20. Each attribute was scored on a hedonic scale from 0 (spoiled) to 3 (fresh), where the limit of acceptance was the score of 2 (black discontinuous line). Samples with scores < 2 were characterized as spoiled, indicating the end of the shelf-life, while samples scoring within the range of 2–3 were labeled as fresh.

Except for the price, consumers tend to purchase products, especially fresh produce, that are macroscopically appealing. Figure 7 shows the effect of packaging on the scores of specific attributes like the odor, color, texture, and freshness of the cherry tomatoes during storage at 15 °C and 25 °C. At 15 °C, tomatoes exposed to carvacrol vapors had the highest scores, at all sensory attributes, followed by PLA and finally by PP samples, on both days that sensory evaluation was performed (Figure 7(I.a),(I.b)). Specifically, tomatoes packaged with the carvacrol-emitting sachet were characterized as acceptable, receiving scores > 2.0 for all sensory attributes on day 20 or close to the acceptance limit for freshness and color on day 40 (Figure 7(I.a),(I.b)). On the contrary, tomatoes packaged in PP were ranked as non-acceptable, receiving scores < 2.0 even from day 20 (avg. scores: odor = 1.9; texture = 1.0; color = 1.8; freshness = 1.3) (Figure 7(I.a)). Regarding storage at 25 °C, PLA-PHB-CARV and PLA samples were still acceptable after 7 days of storage, while PP samples were characterized as spoiled in terms of texture (avg. score: 1.4) and freshness (avg. score: 1.3) (Figure 7(II.a)), since wrinkling was macroscopically observed. It is worth noting that, even after 20 days at 25 °C, tomatoes exposed to carvacrol were organoleptically acceptable regarding odor and color (Figure 7(II.b)). The positive effect of encapsulated or incorporated EOs in edible carriers or sachets, respectively, or their bioactive compounds (carvacrol, thymol) in the sensory quality of tomatoes is also well reported by several previous researchers at different temperatures (8–22 °C) [17,36,42]. Moreover, the panelists noted that no carvacrol was detected during the sensory evaluation. Conclusively, the sensory evaluation results suggest that the use of the carvacrol-emitting sachet would not compromise the consumer’s acceptance, not only when cherry tomatoes were stored at 15 °C, but also until the first 7 days of storage at 25 °C.

### 3.8. Growth of Indigenous Microbiota

**Figure 8:**
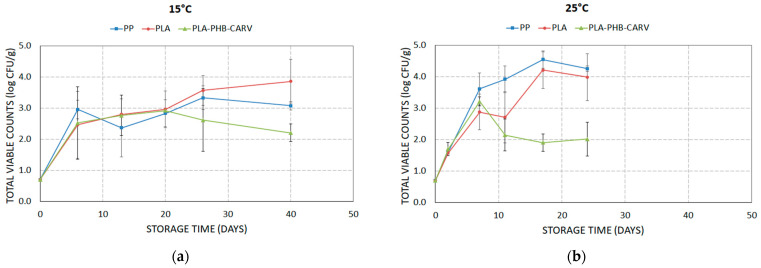
Variation in the total viable counts (TVC) on cherry tomatoes placed in paper kraft trays and packaged in PLA-based EMAP with (PLA-PHB-CARV) or without (PLA) a carvacrol-emitting sachet as well as in macro-perforated polypropylene (PP) bags during storage at (**a**) 15 °C and (**b**) 25 °C.

At 25 °C, the presence of the carvacrol-emitting sachet significantly inhibited TVC in PLA-PHB-CARV samples compared to PLA and PP (*p* < 0.05) (Figure 8b). Specifically, the TVC population of tomatoes packaged in PP and sachet-free PLA-based EMAP reached ca. 4.0 log CFU/g during the 24 days of storage, while on PLA-PHB-CARV samples, the recorded TVC population increased close to 2.0 log CFU/g (Figure 8b). The fact that PLA-PHB-CARV samples presented a delayed ripening process, in terms of % weight loss, TSS, % RI, and texture (as shown in Figure 3, Figure 5 and Figure 6), potentially caused a better cell structure preservation, minimizing any potential leakage of plant cell nutrients, which are considered necessary for the microbial growth, rendering to the reported restricted microbial growth (Figure 8b). On the contrary, at 15 °C, the effect of active EMAP on microbial growth was not that evident (Figure 8a), potentially due to the growth-limiting role of low temperature along with the lower release rate of carvacrol from the sachet [29]. Specifically, TVC increased similarly (*p* ≥ 0.05) among the packaging types during the first 20 days of storage (from 0.7 log CFU/g to ca. 3.0 log CFU/g). Although TVC continued to increase reaching ca. 3.5–4.0 log CFU/g on day 40 in the PP and PLA samples, in PLA-PHB-CARV, TVC remained stable (*p* < 0.05) at ca. 2.0 log CFU/g until the end of the storage at 15 °C. However, it is notable that the level of TVC were below the acceptance limit of 6.0 log CFU/g for mesophilic aerobic bacteria in foods [49], at all studied cases. The availability of the literature results of antimicrobial activity of EOs applied via sachets in tomatoes is limited, and thus the authors conducted comparisons with studies that EOs or their bioactive compounds were applied via other ways with, however, contradictory results, since a series of crucial experimental parameters varied among the studies (storage temperature; type of EO’s application, i.e., coating of fruits, coating the inner side of packaging; mode of action, i.e., diffusion or evaporation). Locali-Pereira et al. (2021) [41] reported that the TVC population of tomatoes packaged in PET boxes coated with a double-layer micro-encapsulated pink pepper EO remained stable, while controls showed an increase of ca. 1.0 log CFU/g during a 21-day period of storage at 25 °C. Pirozzi et al. (2020) [50] reported that tomatoes coated with oregano EO prolonged the shelf life of fruits by reducing the growth of TVC by ca. 4 log CFU/g after 10 days at room temperature compared to controls. However, there are also researchers who have reported their active packaging with an encapsulated mix of oregano EO–cinnamon EO–carvacrol in the inner side of cardboard box, which showed a non-inhibitory effect on the microbial growth during storage at 8 °C [42,51]. Summarizing, the results of the present study indicated that a PLA-based EMAP with a carvacrol-emitting sachet may increase the microbial shelf-life of cherry tomatoes compared to PP and PLA samples by inhibiting the indigenous microbiota even at 25 °C.

## 4. Conclusions

The developed active PLA-based EMAP was studied in the present study as an alternative packaging to prolong the shelf-life of cherry tomatoes during storage at 15 °C and 25 °C. The controlled release of carvacrol through the micro-perforated PHB sachet along with the controlled in-package atmosphere (EMAP) allowed to highly manage the variation in crucial quality parameters like weight loss, decay, TSS accumulation, texture, color, as well as inhibit the growth of indigenous microbiota of cherry tomatoes during storage, not only at 15 °C but also at 25 °C. In that sense, the studied active PLA-based EMAP may be proposed as an advanced alternative packaging versus the existing conventional ones and may be potentially adopted by the fresh-produce industries as a mean of reducing tomato waste from the stage of packaging and during retail distribution. Moreover, the high scores retrieved by the panelists suggest that PLA-PHB-CARV may be highly eligible for commercial purposes. As part of the scale-up process, although Figure 1 presents the commercial version of the active PLA-based EMAP for cherry tomatoes as the authors envisioned and designed it, a feasibility assessment along with a cost analysis should be performed by food industry specialists, taking into account parameters like the increase in functional and raw materials costs related to, i.e., the purchase of PLA and carvacrol as well as the creation of PHB sachet.

## Figures and Tables

**Table 1:** Summary of the information related to specific characteristics per studied packaging type.

Packaging Characteristics	Parameter	Experimental Packaging Type		
		PP	PLA	PLA-PHB-CARV
General characteristics	Container dimensions	22 × 16 × 6 cm	22 × 16 × 6 cm	22 × 16 × 6 cm
	Packaging dimensions	30 × 24 × 6 cm	30 × 24 × 6 cm	30 × 24 × 6 cm
	Weight of tomatoes per package	300 ± 2 g	300 ± 2 g	300 ± 2 g
PHB sachet	Number of holes	N.A. ^1^	N.A.	14
	Diameter per hole	N.A.	N.A.	500 µm
PLA film	Number of holes	N.A.	2	2
	Diameter per hole	N.A.	500 µm	500 µm
PP film	Number of holes	40	N.A.	N.A.
	Diameter per hole	6 mm	N.A.	N.A.

^1^ Non-applied.

**Table 2:** Variation in color difference with true red on cherry tomatoes packaged in micro-perforated PLA bags with (PLA-PHB-CARV) or without (PLA) the addition of a micro-perforated PHB-sachet with carvacrol as well as in macro-perforated polypropylene (PP) bags during storage at 15 °C and 25 °C.

Temperature	Days	PP	PLA	PLA-PHB-CARV
15 °C	0	46.21 ± 2.34 ^Aa^	46.21 ± 2.34 ^Aa^	46.21 ± 2.34 ^Aa^
	6	45.64 ± 1.98 ^Aa^	45.61 ± 1.94 ^ABa^	45.68 ± 2.86 ^BCa^
	13	44.56 ± 2.83 ^Bb^	46.26 ± 1.75 ^Aa^	45.28 ± 2.11 ^BCa^
	20	44.70 ± 2.10 ^Ba^	44.79 ± 2.09 ^BCa^	45.38 ± 1.85 ^Ba^
	26	44.23 ± 3.39 ^Ba^	44.31 ± 2.83 ^Ca^	44.75 ± 2.55 ^BCa^
	40	43.97 ± 2.06 ^Ba^	44.29 ± 2.72 ^Ca^	44.37 ± 2.35 ^Ca^
25 °C	0	46.46 ± 2.28 ^Aa^	46.46 ± 2.28 ^ABa^	46.46 ± 2.28 ^Aa^
	2	46.49 ± 3.43 ^Aa^	47.63 ± 3.13 ^Ab^	46.78 ± 3.59 ^Aab^
	7	44.94 ± 4.00 ^Ba^	47.33 ± 4.60 ^ABb^	47.31 ± 4.02 ^Ab^
	11	44.00 ± 3.45 ^Ba^	46.62 ± 2.80 ^ABb^	45.01 ± 4.20 ^Ba^
	17	43.74 ± 2.96 ^Ba^	44.65 ± 3.03 ^Ca^	46.22 ± 2.80 ^Ab^
	24	43.39 ± 2.44 ^Ba^	46.15 ± 3.75 ^Bb^	46.62 ± 2.78 ^Ab^

Values in the same column within the same packaging type and storage temperature with different superscript uppercase letters are significantly different (*p* ≤ 0.05). Values in the same row within the same storage day and temperature with different superscript lowercase letters are significantly different (*p* ≤ 0.05).

## Data Availability

The original contributions presented in the study are included in the article, further inquiries can be directed to the corresponding author.
